# Cancer-associated fibroblasts as key regulators of lipid metabolism in the tumour microenvironment

**DOI:** 10.1038/s41388-026-03733-9

**Published:** 2026-03-21

**Authors:** Jessamy Adams, Caterina M. Suelzu, Gabriele Strusi, Justin Stebbing

**Affiliations:** Department of Life Sciences, Anglia Ruskin University, Cambridge, UK

**Keywords:** Cancer metabolism, Cancer microenvironment

## Abstract

Alterations in metabolism are recognised as a hallmark of cancer, allowing for rapid proliferation in an environment often hypoxic and short of nutrients. Cells within the tumour microenvironment (TME) often undergo metabolic alterations to adapt to these conditions, and this can also contribute to tumour progression. Cancer associated fibroblasts (CAFs) are amongst the most abundant non-cancerous cells in the TME and the main cells responsible for production and maintenance of the extracellular matrix. However, CAF subtypes can impact tumours in different ways and have been shown to play a role in alterations to lipid metabolism within tumours, being able to produce and secrete lipids, internalise them from the surrounding environment, and undergo fatty acid oxidation. Whilst this is still an emerging area of research, it appears that CAFs can have opposing roles in lipid metabolism in different types of cancer. Understanding the different metabolic pathways utilised in both CAFs and cancer cells and how external factors such as obesity and high fat diets influence them, could provide novel treatment avenues in the future. This review explores the literature surrounding lipid metabolism in CAFs and how this influences tumour progression and treatment resistance.

## Introduction

Tumours are heterogenous ecosystems in which metabolic reprogramming of both the tumour cells and the surrounding microenvironment is necessary to facilitate continued growth [1]. The tumour microenvironment (TME) consists of other cells such as fibroblasts, pericytes, endothelial cells [2], and immune cells including macrophages, neutrophils, B cells and T cells [3], as well as the extracellular matrix (ECM). This is a network of interconnected fibres which surrounds the cells, allowing for transport, communication, and delivery of nutrients. Cancer associated fibroblasts (CAFs) are amongst the most abundant cell types in the TME of many cancers and are the principal producers of the extracellular matrix. CAFs have been shown to contribute to tumour progression in a variety of ways including growth, angiogenesis, metastasis, immune suppression, and treatment resistance [4, 5].

The TME is often a hypoxic and nutrient poor environment that cancer cells adapt to in order to survive and maintain their rapid proliferation. This is overcome by metabolic alterations, one of the hallmarks of cancer [6]. Metabolic reprogramming in cancer was first observed in the Warburg effect. This occurs when cancer cells switch from oxidative phosphorylation to glycolysis for energy, even in the presence of oxygen [7]. Since then, tumours have been found to modify multiple other aspects of metabolism, with lipid metabolic reprogramming also being shown to play a role in progression. Lipids are utilised by cancer cells, not only to provide energy but also as essential ‘building blocks’ in the formation of new cell membranes, and alterations in lipid metabolism are frequently observed in chemoresistant tumours [8, 9]. Tumour cells can fulfil this need in a variety of ways, such as upregulating lipid synthesis, enhancing lipid uptake and storage, and increasing fatty acid oxidation (FAO) [1].

Metabolic reprogramming does not only affect tumour cells themselves; CAFs are increasingly being found to play an important role in the altered metabolism within tumours, potentially contributing to tumour progression [10]. Whilst certain aspects of CAF metabolism have been reviewed elsewhere, their multifaceted role in lipid metabolism is only just being revealed. Therefore, the subject lacks an up to date, comprehensive review which takes into account the effect of external factors such as dietary lipids and obesity. This review aims to address the following three questions: 1. How do CAFs influence treatment response and disease progression? 2. To what extent do dietary lipids and obesity affect the phenotype and functions of CAFs in the TME? 3. What is the current status of pharmaceutical trials on compounds which could target aspects of lipid metabolism?

## CAF origins and heterogeneity

Fibroblasts are typically defined by the expression of markers such as vimentin and platelet-derived growth factor, and by the absence of lineage markers for leukocytes, epithelial, and endothelial cells [4]. In normal physiology, fibroblasts are quiescent and become activated only at sites of tissue injury in response to cytokines such as transforming growth factor β (TGFβ), differentiating into myofibroblasts. This is a contractile phenotype with increased expression of α-smooth muscle actin (αSMA) and production of ECM components that is responsible for wound contraction [11]. Myofibroblasts usually revert to a quiescent state after healing; however, chronic inflammation in an organ can cause them to remain activated, as occurs in cancer.

Many CAFs are thought to originate from resident fibroblasts or stellate cells in pancreatic and liver cancer, which become activated during tumourigenesis [12]. This is supported by findings that normal fibroblasts can become activated to resemble CAFs and support cancer cell growth in vitro via co-culture with cancer cells or tumour conditioned media [13]. However, there is evidence of alternative sources; for example, Quante et al. found that at least 20% of CAFs were derived from mesenchymal stem cells (MSCs) in a mouse model of gastric cancer [14]. MSC-derived CAFs have also been associated with worse prognosis in multiple cancer types inclusing breast cancer [15, 16]. Other identified sources of CAFs include pericytes [17] and cancer cells themselves through epithelial to mesenchymal transition (EMT). This is a process by which cancer cells acquire mesenchymal characteristics with increased migratory capacity [18].

The diverse origins of CAFs are thought to contribute to the heterogeneity observed in CAF populations across numerous solid tumour types (Table 1). This was first observed in pancreatic ductal adenocarcinoma (PDAC), when Öhlund et al. discovered two distinct populations of CAFs, termed myofibroblast CAFs (myCAFs) and inflammatory CAFS (iCAFs). Whilst iCAFs do not express high levels of αSMA, characteristic of activated fibroblasts, they are involved in inflammation within the TME, through the expression of cytokines and chemokines, including interleukins (IL) [12]. The wound-healing aspects of fibroblasts, including ECM remodelling and contractile functions, are thought to be carried out by myCAFs due to their increased expression of αSMA and ECM genes. Some studies, however, have found that these functions are carried out by different subsets of αSMA-positive CAFs, with some classifying matrix-producing CAFs (mCAFs) as distinct from contractile myCAFs [19]. Both myCAFs and iCAFs are thought to be derived from resident fibroblasts and they have been reported to be interchangeable, with different activation pathways [12]. For example, TGFβ signalling increases the proportion of myCAFs, whilst IL-1 increases the proportion of iCAFs via the JAK/STAT pathway [20]. In addition to these two subtypes, Elyada et al. identified an additional population of CAFs expressing MHC class II and CD74, which they termed antigen presenting CAFs (apCAFs) [21]. Whilst myCAFs and iCAFs are both thought to originate from resident fibroblasts, apCAFs have been shown to derive from mesothelial cells in lineage-tracing studies [22]. Vascular CAFs (vCAFs) represent another subtype that may arise from a distinct cellular origin. Their expression of NOTCH3, which is involved in blood vessel formation, together with pericyte markers including RGS5 and MCAM, supports the possibility of a pericyte-derived lineage [23, 24].

**Table 1:** Different subtypes of CAFs.

CAF type	Markers	Proposed origin	Cancer types	References
myCAF	αSMA, FAP	Fibroblasts/PSCs	PDAC, Breast, Lung	[12, 19, 21, 24]
iCAF	IL-6, CD34, CD248	Fibroblasts/PSCs	PDAC, Breast, Lung	[12, 19, 21, 24]
apCAF	CD74, HLA-DR	Mesothelial cells	PDAC, Breast	[21, 24]
vCAF	CD146, MCAM	Pericytes	Breast, Lung	[19, 24]
mCAF	αSMA, MMP11, Collagen	Fibroblasts	Breast, Lung	[19, 24]
Lipid-laden CAF	FASN, SREBP2, ABCA8a	Fibroblasts/PSCs	PDAC, CRC, Breast	[8, 31, 34]
lpmCAF	CD36	Fibroblasts	Hepatic	[44, 45]

The heterogeneity of CAFs contributes to the complex nature of the TME and many studies have so far attempted to define the different subtypes. However, many of these studies categorise them differently, adding to the complexity. Understanding the functions of different CAF subtypes and the variety of ways in which these interact with other cells within the TME is therefore essential in targeting them.

## CAF contribution to lipid metabolism in cancer

CAFs have been shown to contribute to tumour growth and survival by supplying energy sources such as lipids, glucose, lactate, ketones, and amino acids [10]. Previous research has primarily focused on how CAFs can undergo the Warburg effect by increasing glycolysis and secreting lactate, which tumour cells subsequently metabolise via oxidative phosphorylation [25]. However, CAFs have recently been shown to impact lipid metabolism through multiple mechanisms. They can display enhanced lipid synthesis and secretion [26], form lipid droplets, oxidise fatty acids, and induce lipogenesis in cancer cells [27–29]. CAF-produced lipids can promote tumour progression by entering cancer cells through fatty acid transporters [9, 30, 31].

In pancreatic cancers, a substantial proportion of CAFs derive from pancreatic stellate cells (PSCs). These cells store lipid droplets in their quiescent state, which are lost upon activation, including during transition to CAFs [32]. Hossen et al. reported that gold nanoparticles increased lipid synthesis in pancreatic cancer-derived CAFs by upregulating lipogenesis-related genes such as fatty acid synthase (FASN), SREBP2, and FABP3, which they suggested was reversing the CAFs to a quiescent state due to reduced expression of αSMA [33]. However, a lipid-laden CAF subtype has recently been discovered in a SETD2-deficient PDAC murine model, characterised by ABCA8a expression. These CAFs upregulate lipid production and transfer to cancer cells, thereby fuelling oxidative phosphorylation, a phenotype induced by BMP2 signalling from adjacent PDAC cells [34, 35]. Notably, these CAFs lacked αSMA expression, indicating that gold nanoparticles may have altered the CAF subtype rather than restored quiescence.

Conversely, in other cancer types, CAF activation seems to lead to an increase in intracellular lipids. For example, in a murine lung cancer model, accumulation of lipid droplets in fibroblasts increased expression of activated fibroblast markers such as αSMA and FAP, promoting tumour progression [27]. Similar alterations in CAF metabolism were observed in KRAS- mutant colorectal cancers (CRC), where BMP4 and WNT5B secreted by cancer cells drove CAF differentiation into a lipid-rich phenotype that released lipids utilised by tumour cells [36]. BMP signalling has been found to induce myofibroblasts differentiation into adipocytes during hair follicle formation [37], suggesting that cancer cells may exploit this physiological mechanism to obtain lipids.

In addition to providing a source of nutrients for cancer growth, lipids secreted by CAFs appear to directly influence other aspects of cancer progression, including metastasis and angiogenesis. In CRC, lipid-rich CAFs have been found to support cancer progression in two ways. They promote angiogenesis in the TME via expression of vascular endothelial growth factor-A (VEGFA) [36]. Additionally, CAF-derived lipids have been found to play a role in peritoneal metastasis by increasing cancer cell membrane fluidity [38]. In oral squamous cell carcinoma (OSCC), CAF-derived lipids contribute to lipid raft formation in OSCC cells, enhancing migration and invasion [39]. Lipid rafts are cholesterol and sphingolipid enriched areas of the plasma membrane, implicated in EMT, angiogenesis, and metastasis [40]. CAF membranes also contain lipid rafts, which may support metastatic processes. The poor prognosis-CAF marker FAPα has been shown to localise to CAF lipid rafts in vitro, where it contributes to the formation of invadopodia, i.e. cell membrane protrusions that can reshape the ECM and facilitate cell migration [41].

**Fig. 1:**
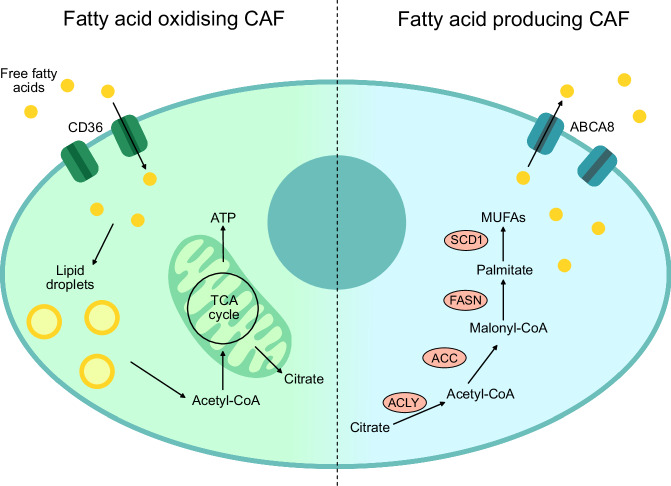
The opposing ways in which CAFs can affect lipid metabolism. CD36-expressing CAFs take up fatty acids from the surrounding environment and store them in lipid droplets. Elevated levels of proteins involved in fatty acid oxidation, such as CPT1, indicate that fatty acids are their primary energy source. In contrast, other CAFs show increased expression of proteins related to fatty acid synthesis, including FASN and SCD1, as well as proteins involved in lipid efflux, such as ABCA8.

One key protein mediating CAF-secreted lipid uptake is the fatty acid transporter CD36, a membrane receptor expressed in multiple cell types and involved in fatty acid uptake and metabolism. CD36 dysregulation is associated with metabolic disorders such as insulin resistance and obesity and has also been linked to cancer metastasis [42, 43]. In CRC, Gong et al. found that lipids secreted by FASN-expressing CAFs are taken up by cancer cells via CD36, promoting metastasis; this effect was attenuated by CD36 inhibition in cancer cells [9]. However, some fibroblast populations also express CD36. In hepatocellular carcinoma (HCC), Zhu et al. identified five different CAF subtypes, including two involved in lipid processing. Lipid processing mCAFs (lpmCAFs) were found to represent an intermediate state between mCAFs and the other subtypes, expressing both lipid metabolism-related proteins, including CD36, and ECM proteins [44]. Uptake of oxidised low-density lipoproteins (LDLs) by these CAFs was shown to trigger the release of migration inhibitory factor (MIF) and promote immunosuppression, which was reduced upon CD36 inhibition. Wang et al. reported that CD36+ CAFs in HCC exhibit increased metabolic activity and lipid droplet formation. They also found that overexpression of CD36 decreased CAF expression of ABCA8, a protein involved in lipid efflux [45], which appears to contrast with the lipid-excreting CAFs observed in PDAC [34]. These findings highlight that different CAF subtypes may play distinct roles in lipid metabolism (Fig. 1). CD36 inhibition in these CAFs concurrently reduced the expression of activated CAF markers such as αSMA and FAP, and suppressed tumour growth [45]. Conversely, in a breast cancer organoid model, CD36+ fibroblasts reduced tumour growth [46], although in vivo relevance remains unclear. In dermal fibroblasts, CD36 expression has been associated with increased FAO and a catabolic phenotype characterised by ECM internalisation and reduced fibrosis [47]. Similar findings in normal fibroblasts from other organs have shown that a decrease in FAO is linked to upregulation of fibrosis-related genes [48], although whether this applies to CAFs remains uncertain. Further research is needed on CD36 expression in CAFs to better understand its impacts on CAF phenotypes.

Taken together, these studies show evidence of varying ways in which CAFs influence metabolism and could provide novel targets for pharmaceuticals. For example, targeting rate-limiting enzymes in the stages of fatty acid synthesis could limit the supply of lipids to tumour cells. Another approach would be targeting fatty acid transporters such as ABCA8 or CD36. However, due to the conflicting roles of CAF subtypes, and the seeming contrasting roles of some transporters such as ABCA8, further research is needed on the mechanisms causing CAFs to adopt these distinct phenotypes. An overview of pharmaceuticals that target different aspects of lipid metabolism is provided in the “Therapeutic applications” section.

## Effects of obesity on CAFs and the TME

The global prevalence of obesity is rising rapidly, with rates projected to reach 25% by 2035 [49]. Obesity has been associated with an increased risk of developing several cancer types as well as with poorer prognosis [50]. There are multiple factors which contribute to this. These include widespread chronic inflammation, increased angiogenesis and dyslipidaemia, which results in elevated levels of free fatty acids [51]. Furthermore, obesity is linked to an increased presence of cancer-associated adipocytes (CAAs), and adipose stem cells (ASCs), which are multipotent stem cells located in adipose tissue [52].

**Fig. 2:**
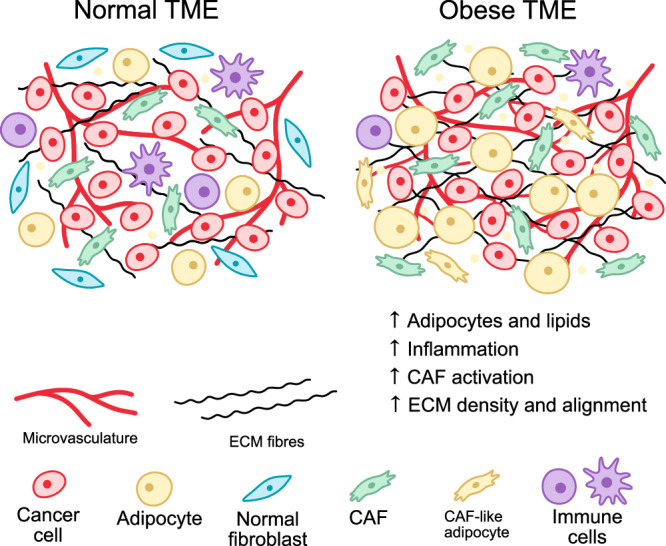
Representation of changes commonly found in the TME of obese tissues. In obesity, tissues exhibit higher levels of free lipids and more numerous, larger adipocytes, as well as an increase in angiogenesis and vessel dysfunction. Adipocytes can also undergo phenotypic changes into cells which resemble CAFs in marker expression and function. In addition to this, there is an increase in CAF activation via inflammatory mediators such as TGFβ. This increased CAF activation also leads to greater ECM deposition and crosslinking and contributes to reduced antitumour immunity.

In addition to CAFs acquiring an adipose-like phenotype, ASCs and CAAs, can serve as a source of CAFs, particularly in adipose tissue-rich cancers such as breast cancer. This transformation occurs more frequently in ASCs from obese patients compared with those from lean patients [53]. Moreover, CAFs derived from overweight or obese individuals exhibit a higher lipid content than those from patients of healthy weight [54]. Obesity also promotes inflammation, leading to excessive production of inflammatory cytokines and chemokines, including TGFβ and IL-1. which stimulate the differentiation of myCAFs and iCAFs, respectively [20]. This could potentially increase CAF activity within the TME, which is associated with poorer prognosis (Fig. 2).

A well-established link exists between obesity and fibrosis in both healthy and tumour tissue, likely driven by enhanced fibroblast activation. Obesity has also been shown to influence fibroblast subtypes. Adipose tissue from obese mice contains more myofibroblasts than that from lean mice, which has been linked to production of a denser and more aligned ECM [55]. Interestingly, Ritter et al. reported that ASCs from obese breast cancer patients expressed higher levels of myCAF markers, whereas ASCs from lean patients could also differentiate into CAFs, but exhibited lower αSMA expression and increased expression of iCAF markers [56]. These findings correlate with the work of Lo P-K et al., which showed that breast cancer-derived CAFs from obese mice expressed higher levels of ECM-related genes, such as collagens and elastin, and lower levels of immune-regulatory genes compared with CAFs from lean mice [57].

Obesity has further been associated with increased metastatic rates across multiple tumour types. Hillers-Ziemer et al. investigated this correlation in a mouse model of breast cancer and found that lung fibroblasts from obese mice became activated similarly to CAFs, producing greater amounts of ECM components, such as collagen, alongside proinflammatory cytokines. These fibroblasts facilitated the invasion of bone marrow-derived myeloid lineage cells via colony-stimulating factor 2 into the lung, creating a pre-metastatic niche that promoted lung metastasis [58].

Obesity causes numerous changes to the phenotype of multiple cells within the TME including CAFs and adipocytes, as well as the ECM and vascularity. Due to the increasing prevalence of obesity, and the increased risk of cancer in these individuals, it would be beneficial to identify treatment options that take into account these differences in the TME.

## Effects of different fatty acids

High fat diets have been linked to cancer progression and worse prognosis although there is little research about their impact on CAFs. Evidence suggests that high fat diets may promote the differentiation of other cell types into CAFs. For example, colonic MSCs from mice fed a high fat diet exhibited characteristics more similar to CAFs, compared with those fed a normal diet [59]. High fat diets have also been found to affect CAF gene expression in breast cancer. Li et al. recently found that high fat diets decreased CAF expression of PLAT, which encodes tissue-type plasminogen activator protein, in a murine model of breast cancer [60]. This reduction allowed for increased polarisation of M2 macrophages, promoting cancer cell migration and invasion.

Specific fatty acids have been found to impact the TME [61]. Fatty acids are broadly classified into saturated, monounsaturated or polyunsaturated, but even within the same category, their effect can diverge. For example, palmitic acid has been found to contribute to increased CRC development, whereas stearic acid alone does not exhibit similar effects, despite them both being saturated fatty acids [62]. Interestingly, stearic acid has been reported to attenuate breast cancer progression [63]. Deng et al. found that palmitic acid specifically activated the NF-κB pathway in CRC, which in turn induced fibroblasts activation into an mCAF phenotype. These mCAFs produced a stiffer and more aligned ECM which can lead to obstruction in CRC and is associated with worse outcomes [64].

Unsaturated fatty acids include ω-6 and ω-3 polyunsaturated fatty acids and ω-9 monounsaturated fatty acids. Koundouros et al. demontrated that ω-6 linoleic acid (LA) could activate the mechanistic target of rapamycin complex-1 (mTORC1) via FABP5 in triple negative breast cancer (TNBC). This was found to increase tumour growth in a mouse xenograft model fed a diet high in ω-6 LA compared with one enriched in ω-3 linolenic acid (ALA) [65]. Conversely, ω-3 fatty acids are generally considered to be beneficial in cancer, for example via mitigation of chemoresistance [66]. In CRC, ω-3 ALA has been shown to reverse chemoresistance in CRC by decreasing CAF activation, especially FAP+ CAFs [67]. This effect occurs through inhibition of SOX2, which downregulates osteopontin, a known CAF activator across multiple cancer types [13, 68]. ω-3 fatty acids have also been implicated in reducing CAF-mediated angiogenesis. Taguchi et al. investigated this using a mouse model of lung cancer capable of converting ω-6 to ω-3 fatty acids. CAFs in this model exhibited reduced MMP-7 levels, a key factor involved in angiogenesis [69]. Additionally, Ando et al. found that eicosapentaenoic acid, another ω-3 fatty acid, reduced expression of IL6 and VEGF in vitro [70].

Whilst ω-3 and ω-6 have well established roles in cancer, studies involving ω-9 fatty acids, the most common of which is oleic acid, have yielded conflicting results [71]. Oleic acid, the main component of olive oil, is thought to have anti-inflammatory effects [72] and has been found to induce apoptosis and reduce tumour growth [73]. However, oleic acid has also been found to increase angiopoietin-like 4 (ANGPTL4) expression in head and neck squamous cell carcinoma (HNSCC) and CRC, which promotes MMPs expression, metastasis [74] and anoikis [75]. Oleic acid derived from CAFs has been shown to increase stemness and lipid synthesis in cancer cells via the upregulation of stearoyl-CoA desaturase (SCD) and sterol regulatory-element binding protein (SREBP1) in cancer cells [76]. SCDs catalyse the formation of monounsaturated fatty acids from saturated fatty acids and SCD1 has been associated with CAFs. In breast cancer, CAFs were found to upregulate SCD1 expresion in cancer cells, increasing membrane fluidity and migration [77]. Oleic acid was found to have similar effects even when SCD1 was inhibited [78].

This evidence suggests that dietary interventions alongside traditional therapy could improve outcomes. For example, increasing beneficial fatty acids such as stearic acid and ALA, or reduction of detrimental fatty acids such as palmitic acid and LA, may improve outcomes. However, due to the research largely involving murine models, it is unclear to what extent this would translate to human tumours. It may be more effective to target pathways activated by different fatty acids directly. However, more research would be useful in order to better characterise these targets.

## Therapeutic applications

Several strategies have been explored to target CAFs in cancer, often with conflicting results. For example, in murine models of PDAC, targeting αSMA + CAFs led to more aggressive, undifferentiated tumours with increased hypoxia [79]. Another study reported that targeting αSMA + CAFs increased tumour progression, whereas targeting FAP+ CAFs produced the opposite effect [80]. These findings show that certain CAF subtypes may have a tumour suppressive effect. Therefore, a deeper understanding of CAFs subtypes and the specific pathways mediating their pro- and anti- cancer effects is required for identifying more effective therapeutic targets. Given the diverse roles of CAFs in lipid metabolism within the TME, targeting specific elements of these pathways may prove effective in cancer treatment (Table 2).

**Table 2:** Treatments which potentially target aspects of lipid metabolism in cancer.

Target	Function	Treatment	Phase	Combination	Cancer type	Reference
ACLY	Production of actetyl-CoA	SB-204990	In vivo	None	Lung/PCa	[80]
			In vivo	Vemurafenib	Melanoma	[81]
		Potassium hydroxycitrate	In vivo	Vemurafenib	Melanoma	[81]
ACC	Conversion of acetyl-CoA to malonyl-CoA	ND-646	In vivo	Carboplatin	NSCLC	[82]
		ND-654	In vivo	Sorafenib	HCC	[83]
FASN	Synthesis of palmitic acid	Denifanstat	Phase I	Taxane	Multiple	NCT02223247 / [84]
			Phase I	Enzalutamide	Prostate	NCT05743621
			Phase II	Bevacizumab	Astrocytoma	NCT03032484 / [85]
			Phase II	Taxane / trastuzumab	Breast (HER2 + )	NCT03179904
			Phase III	Bevacizumab	Glioblastoma	NCT05118776
		Orlistat	Phase II	Osimertnib	Lung	NCT06818955
			In vivo	None	Prostate	[86]
SCD1	MUFA synthesis	MTI-301	Phase I	None	Multiple	NCT06911008
			In vivo	Sorafenib	HCC	[7]
SREBP1	Regulator of lipid synthesis	Fatostatin	In vivo	Progesterone	Endometrial	[87]
HMGCR	Cholesterol synthesis	Simvastatin	Phase II	Cyclophosphamide/Adriamycin/	Breast (locally advanced)	NCT04418089 / [89]
				Fluorouracil		
			Phase II	Gemcitabine	Pancreatic (advanced)	NCT00944463 / [90]
			Phase III	Capecitabine-cisplatin	Gastric	NCT01099085 / [91]
		Pitavastatin	Phase I	Temozolomide	Glioblastoma	NCT05977738
			Phase II / III	Doxorubicin / cyclophosphamide	BC	NCT04705909 / [92]
CD36	Fatty acid transporter	JC63.1	In vivo	None	CRC	[8]
			In vivo	None	HNSCC	[42]
		FA6.152	In vivo	None	HNSCC	[42]
		PLT012	In vivo	Immunotherapy	Liver	[93]
CPT	Rate limiting enzyme of FAO	Etomoxir	In vivo	Anti-VEGF antibody	Liver	[97]
			In vivo	Radiotherapy	Nasopharangeal	[98]
		Perhexiline	In vitro	None	Ovarian	[99]

### Targeting lipid synthesis

One of the major ways CAFs have been found to contribute to cancer metabolism is by secreting lipids into the TME. Therefore, inhibiting enzymes involved in fatty acid synthesis represents a potential therapeutic strategy. Inhibition of multiple stages of this process are currently being investigated for cancer treatment. The first step involves the production of acetyl-CoA, catalysed by ATP citrate lyase (ACLY) before its conversion to malonyl-CoA by acetyl CoA carboxylase (ACC). SB-204990, an ACLY inhibitor, has been shown to reduce tumour growth both in vitro and in vivo [81]. Furthermore, ACLY inhibition with either SB-204990 or potassium hydroxycitrate increased sensitivity to vemurafenib in a melanoma mouse model [82]. ACC inhibitors, such as ND-646 and ND-654, have demonstrated promising results in mice [83, 84]; however, neither these nor the ACLY inhibitors have progressed to clinical trials. FASN, which is highly expressed in certain CAF subtypes, catalyses the subsequent step, producing palmitic acid from malonyl-CoA. TVB-2640 (also known as ASC40 or Denifanstat), a FASN inhibitor, has completed phase I and II clinical trials [85, 86] and is currently in phase III (NCT05118776). Additionally, orlistat, a weight-loss drug, has been shown to inhibit FASN in cancer [87]. SCD1 catalyses the conversion of palmitic acid into monounsaturated fatty acids such as oleic acid and is also expressed in some CAF subtypes. Multiple SCD1 inhibitors have been tested preclinically, and MTI-301 has recently entered a phase I clinical trial (NCT06911008).

Beyond direct inhibition of fatty acid synthesis, other studies have focused on targeting the upstream regulation. SREBP1, an upstream regulator of lipid synthesis, can be upregulated in cancer cells by CAF-derived oleic acid [76]. Its inhibition with fatostatin reduced CAF-induced expression of SCD1 in breast cancer cells [77]. Fatostatin has also been shown to improve treatment efficacy in progesterone-resistant endometrial cancer in vivo, suggesting a promising approach for overcoming resistance [88].

### Targeting cholesterol synthesis

Another approach involves targeting cholesterol biosynthesis, which contributes to membrane fluidity and has been linked to metastasis [89]. Although research on cholesterol metabolism in CAFs is limited, Neuwirt et al. demonstrated that prostate cancer CAFs contribute to cancer progression by upregulating cholesterol and steroid production in tumour cells using a coculture spheroid model. Treatment of these spheroids with simvastatin, an inhibitor of cholesterol and steroid synthesis, significantly reduced their growth [89]. Statins lower cholesterol by inhibiting 3-hydroxy-3-methylglutaryl-CoA reductase (HMGCR), a key enzyme required for its production, and are widely used to treat hypercholesterolaemia. They are also being investigated for cancer therapy; however, clinical trials of simvastatin have not shown significant improvements in response [90–92]. Pitavastatin has demonstrated promise in one clinical trial, but the sample size was limited, warranting further investigation [93].

### Targeting fatty acid transport and oxidation

Transport of CAF-derived lipids into cancer cells occurs via specific transport proteins; blocking these could limit energy availability and slow tumour growth. One of the major proteins involved in fatty acids uptake is CD36 [42]. Monoclonal antibodies against CD36, such as FA6.152 and JC63.1, have reduced CAF-induced migration in vivo [9, 42]. A humanised anti-CD36 IgG4 antibody has recently been tested in mice and cynomolgus monkeys and is expected to enter clinical trials soon [94]. Inhibition of CD36 may have a dual effect, as some CAFs also express this protein [45]; however, further research is needed to clarify its impact on CAFs. None of these CD36 inhibitors have yet reached clinical trials, although pitavastatin has been suggested to inhibit CD36 in vivo [95].

Another potential target is ABCA8, a transporter responsible for lipid efflux from CAFs into the TME in pancreatic cancer [34]. Overexpression of ABCA8 has been associated with gemcitabine resistance in pancreatic cancer, both in vivo and in vitro [96]. Nonetheless, ABCA8 acts as a tumour suppressor in other cancers, such as prostate cancer, where its upregulation inhibited tumour progression [97].

FAO is the initial step in breaking down fatty acids for energy and is utilised by both cancer cells and CAFs. Carnitine palmitoyltransferase 1 (CPT1) is the rate-limiting enzyme in FAO, and increased expression, in either CAFs or cancer cells, correlates with cancer progression and poor prognosis [28]. Etomoxir, the most studied CPT1 inhibitor, has been found to resensitise tumours to anti-angiogenic treatment and radiotherapy in vivo, but has not progressed to clinical trials [98, 99], a feature of many compounds in this space. Other studies have explored repurposing existing drugs, such as perhexiline. This is a CPT1 inhibitor used to treat angina, but has recently been shown to be effective in a 3D microfluidic tumour model comprising cancer cells and fibroblasts. Interestingly, etomoxir did not induce cell death in this model [100].

## Conclusions and future perspective

CAFs exhibit a multifaceted role in lipid metabolism within the TME. Distinct CAF phenotypes can exert opposing functions, increasing either lipid uptake and FAO or lipid production and secretion. Consequently, targeting specific elements of lipid metabolism in CAFs and their interacting cells provides new avenues of treatment. However, it is necessary to further understand the variety of CAF subtypes and the mechanisms which lead to the formation of CAFs with pro- or anti-tumour functions. Likewise, some lipid-mediating proteins appear to display context-dependant roles. For example, ABCA8, which has been found to have either tumour suppressing or chemoresistance functions. This could be due to differences in the models used to study the TME. In vitro models cannot fully recreate the complexity of the TME, however, 3D models such as spheroids and organoids provide greater accuracy than traditional 2D culture methods. Additionally, in vivo murine models have been frequently used to study lipid metabolism in tumours. These are beneficial as they allow for study of whole tumours, however, murine gene functions might not accurately reflect human genes and therefore provide conflicting results.

Pre-existing metabolic disorders such as obesity and dyslipidaemia must be considered due to their effect on the activation and differentiation of CAFs, creating an inflammatory and fibrotic TME. Furthermore, fatty acid composition has been shown to modulate the TME and key aspects of cancer progression such as metastasis. Dietary strategies, such as reducing the intake of fatty acids linked to poor outcomes, such as ω-6 linoleic acid, or supplementation with those with demonstrated benefits, such as ω-3 ALA, could provide clinical advantages, but this has yet to be shown in prospective randomised studies. However, the exact mechanisms through which these interventions impact the TME, especially in the context of CAFs, are largely unknown and therefore warrant further investigation.

It is also important to take into account the wider context of the TME. Due to the wide ranging effects of CAFs on lipid metabolism in the TME, it seems likely that this would impact the metabolic pathways in all other cells in the TME. However, current studies have focused mainly on the alterations to cancer cell lipid metabolism. The effect CAFs have on lipid metabolism in other cell types, particularly immune cells, is an area which warrants more research.

Alterations in lipid metabolism in cancer are particularly relevant to treatment resistance, as upregulation of lipid metabolism is frequently observed following chemotherapy. Therefore, co-administration of inhibitors of fatty acid synthesis, uptake or processing alongside chemotherapy may represent a viable method of overcoming chemoresistance. However, only a limited number of these treatments have so far reached clinical trials in the context of cancer, with inconsistent results. In conclusion, whilst this is a promising area of research with the potential to identify novel therapeutic strategies which, in combination with existing chemotherapies, could mitigate resistance, further investigation is necessary to elucidate the complexity of metabolic pathways and how this is impacted by intercellular crosstalk within the TME.
